# Psychological distress in the neonatal intensive care unit: a meta-review

**DOI:** 10.1038/s41390-024-03599-1

**Published:** 2024-09-26

**Authors:** Lizelle van Wyk, Athenkosi P. Majiza, Cordelia S. E. Ely, Lynn T. Singer

**Affiliations:** 1 Department Paediatrics and Child Health, Stellenbosch University, Cape Town, South Africa; 2 Departments of Population and Quantitative Health Sciences, Pediatrics, Psychiatry and Psychological Sciences, Case Western Reserve University School of Medicine, Cleveland, OH USA

## Abstract

**Introduction:**

Parental psychological distress (PD) (anxiety, depression, stress and post-traumatic stress syndrome) can adversely affect parents’ own physical and mental health as well as their children’s long-term health and development. Numerous studies have addressed PD in mothers of infants admitted to NICU, with interventions proposed, but few have addressed the impact on fathers or other family members. The present review examined systematic reviews that addressed PD in NICU and potential interventions.

**Methods:**

A meta-review was performed by searching various databases between 2000 and May 2024.

**Results:**

Fifty-four studies were included. The incidence of maternal PD varied depending on the screening tool used (13–93%), as did paternal PD (0.08–46%). The incidence of PD in sexual, racial and gender minorities, siblings, grandparents and those in lower-middle income countries is not known. Numerous screening tools were used with a wide variety of cut-off values. Various intervention programmes were evaluated and showed contradictory evidence regarding their effect on PD.

**Discussion:**

Routine screening should be implemented together with a combination of interventional programmes, specifically family-centred interventions. More research is required for PD in siblings, sexual and gender minority parents as well as parents living in low middle income countries.

**Impact statement:**

- Psychological distress is high in NICU, affecting parents and siblings.
- Maternal psychological distress may have long lasting effects on infant health and differs from that of fathers, who require as much attention as mothers
- Little is known about emotional stress in siblings and sex and gender minority group peoples
- Few interventions showed conclusive effectiveness in reducing psychological distress with combination interventions showing more effectiveness than single interventions

## Introduction

Giving birth to a healthy infant is the expectation of most parents,^1,2^ but the admission of a preterm or sick newborn infant to the neonatal unit (special care infant unit or neonatal intensive care unit (NICU)) shatters that expectation.^3^ While intensive care may ensure the infant’s survival, parental and family experiences of the NICU are often experienced as traumatic.^4^

NICU admissions have increased 20–30% despite decreasing birth rates,^5,6^ leading to an increasing number of parents exposed to the stress of NICU. The perinatal incidence of depression and anxiety in parents is 1–3%,^7^ but this may increase dramatically after their infant is admitted to NICU.^8^ Parental psychological distress (PD) entails anxiety, depression, stress and post-traumatic stress syndrome (PTSD). These mental health disorders have significant effects on parents’ general health and long-term mental health^9^ but have also been shown to adversely affect their child’s long-term health and development.^10^ Thus, early identification and management of parental PD in NICU is important to their infant’s recovery.

With the admission of an infant to NICU, the entire family unit may be affected but most research in this field addresses the incidence and management of PD of the mother^11,12^ whilst less evidence is available for both parents^13,14^ and fathers.^15,16^ No data are available addressing PD for other family members or types of families.

Various reviews of parental PD in NICU have been performed since the 1980’s, describing the incidence, screening, prevention and management of PD. As the management and survival of preterm and sick infants has significantly improved since then, there have been major paradigm shifts in neonatal management since the 2000’s, encompassing more holistic views of family engagement. Most reviews have concentrated on the incidence, screening and/ or management of mothers, fathers or parents but none have focused on the family as a whole. The admission of an infant to NICU affects the entire family, and a global understanding of the PD experienced by the entire family is required in order to adequately support the entire family during this stressful period.

This review aimed to provide a comprehensive summary of the incidence of psychological distress (PD) (stress, anxiety, depression and post-traumatic stress disorder (PTSD)) in all family members (mothers and fathers of all genders, siblings, and grandparents) with an infant admitted to the NICU. The review also aimed to provide a comprehensive summary of currently available therapeutic interventions aimed at the prevention and/ or management of PD in all family members with an infant admitted to the NICU.

## Methods

A meta-review approach was used to determine the psychological effect of NICU on all family members. Databases searched included PubMed, Scopus, PsycINFO, CINAHL and Web of Science, restricted to articles between 2000 and May 2024. Only systematic reviews, of any type, were included. EndNote and Rayyan were used for screening and article management. Studies were screened based on title and abstract for inclusion and exclusion criteria by 2 reviewers (LVW, LS). Families were defined as mothers and fathers (of any gender), siblings and grandparents. PD was defined as stress, depression, anxiety and PTSD. Articles describing incidence, screening or diagnostic methods, prevention or therapeutic interventions, in family members whilst the infant was admitted to NICU, were included. Time range (2000–2024) was used as this represented a time period of increased PD screening in mothers, with increasing inclusion of other family members, and high quality neonatal medical care. Qualitative and quantitative reviews were included. Data after infants and/or family members were discharged from NICU were excluded. Studies were also excluded if they only referred to attachment, bonding, coping strategies, quality of life or sleep parameters. COVID studies and studies regarding the death of an infant were excluded as they represent an increase above the normal distress status. Remaining articles were screened as full articles. AMSTAR2 was used for quality appraisal of included reviews.

Included reviews were divided into those of a diagnostic nature (providing incidence and risk factors for PD) and those of a therapeutic nature (providing PD data after an intervention), based on the primary aim or objective stated in the study. Quantitative data were mostly reported as standardised mean difference, but in various ways. Qualitative data were summarised into themes or metaphors, using different analytical methods.

A summary of the included reviews can be found in supplementary Tables 1 and 3 (quality of diagnostic and therapeutic systematic reviews, respectively) and 2 and 4 (results from diagnostic and therapeutic systematic reviews, respectively). Most reviews provided sufficient details regarding search strategy, inclusion and exclusion details. The majority of studies also performed quality appraisals and bias analysis, although a variety of tools were used. Heterogeneity in all studies was high due to the variety of diagnostic tools for PD, the variety of cut-off scores used to diagnose PD and the multitude of interventions available.

## Results

**Fig. 1:**
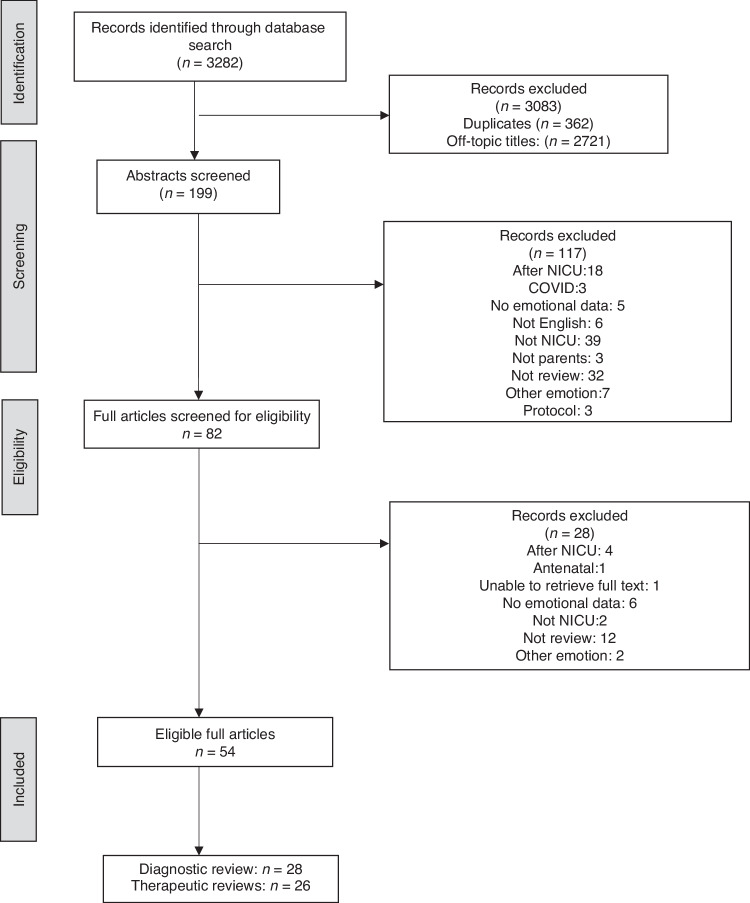
Flow diagram for search strategy and study selection.

Database search delivered 3282 articles, which after exclusions, left 199 articles. Eight-two articles were eligible for full screening and a further 28 were excluded as they had already been incorporated in reviews (Fig. 1). Fifty-four studies were included in the final review. Of these, 26 studies provided diagnostic results – quantitative and qualitative data regarding PD. A further 28 studies provided qualitative and quantitative data regarding the outcomes of therapeutic interventions. Two diagnostic studies also provided narrative reviews of therapeutic interventions.

### The NICU environment as a stressor

The NICU environment itself is alien and stressful to most parents, consisting of unusual lights, periods of unfamiliar lighting (low or minimal), unfamiliar noise patterns and repeated disruptions due to procedures that interfere with parent‒child interaction.^17,18^ Additionally, the immature physical characteristics of preterm infants in the NICU may worsen parental distress and may increase their doubts about their infant’s chances of survival.^19^

Various factors associated with NICU (sights and sounds, baby looks and behaviours, parental relationship with baby and parental role alteration and communication with staff) may affect the parents’ PD.^20^ Maternal PD, regarding the physical NICU environment, is primarily related to her relationship with the baby (separation, fear, hopelessness) as well as her parental role (lack of bonding, inability to feed baby), with sights and sounds being less of a stressor.^20^

Although paternal PD has been noted to be similar in terms of the relationship with the baby, stressors regarding the paternal PD are primarily due to the lack of bond and lack of visiting access to their baby.^20^ This difference may be accounted for by the father usually returning to work after the birth whilst the mother remains with the infant. Stressors may also change over time as parents accommodate to the sensory stimuli of the NICU and changes in parental role whilst later stress may be due to infant behaviours and appearance.^21^ In some countries, sights and sounds may be more associated with parental PD than a change in parental role, which may possibly be explained by low parental education levels.^22^

### Maternal psychological distress

Motherhood is often a societal expectation.^23^ The difference between maternal expectations and the reality of the pregnancy outcome, an unexpected NICU admission, may be experienced as a traumatic life event^3,24^ and may negatively affect maternal mental health.^25^ The incidence of maternal PD has been shown to vary widely: anxiety, 13–93%; depression, 18–52%; stress, 23–76%^26^ and PTSD, 4.5–79%,^6,24^ with rates varying depending on the screening tool used.^6,27^ Maternal PD is also greater than that of fathers^17^ and higher in mothers with preterm infants compared to mothers with term infants.^17^

Stress, anxiety, depression and PTSD often overlap, but are often only identified as single entities, whilst, in reality, being a spectrum of disorders.^6^ Risk factors and contributing factors overlap significantly.^25^ Causes for maternal distress have been shown to differ from those for a father^1,4,17,20–22,28–31^ but many similarities are also present. Causes for maternal PD occur at various levels, extending from personal, pregnancy and infant related, maternal mental health history and family support structures to institutional factors.^29,30^

Maternal demographic factors have shown variable association with PD levels: advanced maternal age,^1,17,20,30^ low education and occupation,^17,20,30^ race,^1,32^ history of mental illness,^17,30^ low socio-economic status,^30,32^ relationship status,^20,21,30^ religion,^20^ travel distance from hospital,^6^ maternal personality^33,34^ and interpersonal violence.^35^ Pregnancy related factors include parity/ birth order,^20^ unplanned pregnancy^36^ and caesarean section delivery.^6^ Infant demographics have shown variable effects on maternal PD: sex,^21,30^ birth weight,^21,30^ gestational age,^21,30^ illness severity and length of hospitalisation.^21,30^ Complicated childbirth and requiring neonatal transport to an NICU may further increase maternal PD.^17,30^ Separation from her infant may be the dominant reason for PD in many mothers.^20,21,28^ A lack of information from medical personnel^21^ and the perceived negative attitudes of medical personnel may further increase maternal PD.^21^ The risk of PTSD increases significantly with pre-existing mental health disorders, depression and anxiety in the NICU, a caesarean section delivery and a lower education.^30^

The sounds, lights and technology in the NICU preventing access to their baby,^1,31^ the inability to prevent their infant’s pain, limited ability to care for the infant and feelings of helplessness have all been shown to further contribute to the initial PD in the mother.^18,31^ The effects of the physical NICU environment differ between mothers, depending on geographical location.^22^ These factors contribute to the experience of an “altered parental role” for mothers, which they experience as highly distressful.^18,20,22,28,31,32^ Feelings of uncertainty, disconnection from their infant, lack of confidence as a mother, further compound their PD.^28,37,38^ Adolescent mothers with an infant in the NICU may experience greater PD and require additional support.^35^

Mothers remain anxious about the survival of their preterm infant, and their ability to manage the situation and care for their infant upon discharge.^31^ Emotions do progress throughout the NICU stay, with some stressors decreasing (noise, lights and baby’s appearance, struggling to become a mother) whilst others may increase (anxiety regarding caring for the infant at home).^4,18,38^ Maternal PD is also associated with paternal PD, and vice versa.^8^

### Paternal psychological distress

Paternal PD has generally been noted to be less severe than in mothers, but also varies widely: anxiety 0.09–46%, depression 0.08–18%, stress 0.06–35%^25^ and PTSD 0–33%,^26^ also depending on the screening tool used.^25^ Despite lower rates of symptomatology, fathers have also described the NICU as an “emotional roller coaster”^26^ and a traumatic experience.^39^ Fathers are often the first to see the sick infant, as the mother is still recovering, and they may be more concerned about the mother’s well-being than that of the infant.^17,40^ Paternal care may therefore extend to providing emotional support to a distressed mother^16^ as well as the sick infant.^16^ This may cause them to feel torn between responsibilities, resulting in increased PD.^39^

It is important to understand the gender differences in the presentation of PD in NICU. In most societies, fathers are considered breadwinners, authoritarians and the protectors of family members,^16^ although roles may differ between cultures.^41^ In contrast to mothers, who often present with anxiety, depression and PTSD, fathers are more likely to exhibit fatigue, irritability, social withdrawal, escapist activities and display hostility and anger.^42^

Paternal demographic variables associated with PD may differ from those in mothers. Similar to mothers, fathers of preterm infants have higher PD than those of term infants.^40^ Younger paternal age, an infant’s extreme prematurity and extremely low birthweight may increase paternal PD whereas being married may decrease it.^40^ Other variables that have shown differences from maternal stressors are employment status, previous pregnancies, infant’s gender, education level, and income level.^20,25,31,43^

The change in parental role is also one of the most stressful factors for fathers.^40^ Fathers also often experience the NICU as an unfamiliar environment^39^ where they are subservient to the mother,^44^ are not involved infant care,^16,44^ nor involved in communication or decision-making.^40^ The need to balance work and life^39,40^ and to remain strong^16^ can lead to feelings of vulnerability^44^ and isolation,^39^ thereby increasing their PD. Paternal stressors may also relate to financial costs,^20^ various home matters,^41,45^ lack of family support,^16^ being required to fulfil dual roles of mother and father,^28^ need for active engagement in care and decision-making, feelings of helplessness to protect their infant from pain^16^ and a need to be considered as a parent rather than only as maternal support.^16^ Although stressors may differ between mothers and fathers, fathers also experience similar emotional changes as the NICU journey progresses.^4,38^

Given the historical masculine-gendered role of fathering, it is perhaps not surprising that fathers become distressed when not ‘in-charge’ and not able to be their infant’s protector.^40^

### Minority group parents’ psychological distress

Minority stress is well known among lesbian, gay bisexual, transgender, queer, intersex, asexual and more identities peoples (LGBTQIA + ), grouped as sexual and gender minorities. Avoidance and distrust of healthcare, pre-conception depression, and socio-economic barriers may increase stress in LGBTQIA+ pregnant peoples.^46^ Postpartum anxiety is known to be increased in sexual minority peoples^47^ and both anxiety and postpartum depression are higher than in heterosexual women.^48^

Little is known about how admission of an infant to NICU affects LGBTQIA parents’ PD. Minority parents may be faced with discrimination and not be treated as a parent due to their non-biological state.^49^ Co-parents require the same respect, support, collaboration, involvement, information sharing and empowerment as that provided to heterosexual parents.^49^ The PD of the second parent should also be kept in mind as their PD may differ to that of the primary parent.^50^ The different needs of gay fathers, lesbian co-mothers and other minority group parents should be recognised.^50^

### Sibling and grandparents’ psychological distress

Little is known regarding siblings’ emotional reactions to a new sibling admitted to NICU and the incidence of sibling PD is also unknown. Siblings may experience the birth of their new sibling intensely.^51^ The family stressors of having an infant in the NICU may lead a sibling to develop various emotional responses, including anxiety, jealousy of parents, excitement or feeling abandoned.^52^ These in turn may lead to acting up, becoming quiet and withdrawn, difficulties eating and sleeping, regressing into infantile behaviours (thumb sucking, bed wetting, etc) or developing trouble at school.^52^

Dependant on age, the sibling’s “NICU experience” may be positive or negative. School-aged siblings have a better understanding of the meaning and implications of a prematurely born infant with a prolonged hospitalisation^53^ whereas younger siblings may question the existence of the new sibling, increasing their PD.^52^ Siblings of infants in NICU are often faced with the increased absence and decreased attention from parents due to their sibling’s competing demands of time and attention.^52^ Maternal attention may be distracted due to her own PD and preoccupation with the infant in NICU, leaving the older sibling reliant on already worried and anxious parents.^51^ Siblings may become afraid of losing their parent’s love, ambivalent towards their parents, jealous of their new sibling, insecure due to changes in family life, as well as frustrated or sad at having to leave their NICU sibling in a hospital.^52^

Sibling care is often shifted onto the father, grandparents and extended family, which can lead to feelings of displacement, further worsening the sibling’s PD.^54^ Younger siblings are often not allowed to visit the NICU, and older siblings may be frightened by the environment,^55^ increasing their sense of isolation.

No data is available describing the incidence of PD in grandparents with an infant in NICU. However, grandparents are often regarded as a support system during periods of crisis. Grandmothers also experience PD and anxiety with a grandchild in NICU, but this may be tempered by feelings that the parents require support, requiring the grandparents to fulfil a new role.^56^ Grandmothers can also be helpful in decreasing maternal depression by providing support and information.^57^

### Differences in parental psychological distress in high vs. low resource settings

In high resource countries, the prevalence of maternal mental health disorders varies between 5 and 24%, whereas the incidence in low resource countries may be as high as 50%.^58^ Little is known about differences in paternal mental health in high vs. low resource countries. Mothers in low resource settings are disproportionately exposed to various cumulative risk factors.^59^ Poor health literacy regarding a high-risk pregnancy and preterm birth and poor utilisation of healthcare remains high in low resource settings.^60^ As a result of the continued discrepancies in maternal mental health outcomes, parental PD is increasingly being incorporated into global health research, especially in low resource settings.^61^

In many low resource settings, there is a high burden of preterm births. Combined with this there are language barriers,^20^ lack of paid maternity or paternity leave, far travel distances to hospital, lack of or costly transportation, and childcare issues^38,62^ which further contribute to PD in low resource parents. Many NICUs in these environments remain technology- and provider-centred, with little parental engagement,^63^ further adding to parental PD.

There is also evidence that the pattern of parental PD differs between high and low resource countries, with stressors differing on multiple levels.^22^ In low resource countries, a preterm infant’s medical stability may be of more concern to mothers than long term developmental outcome.^64^ Cultural traditions and beliefs influence and are influenced by an infant’s prematurity.^64^ In India and Africa, parents and family members may be concerned about missing cultural ceremonies and traditional practices after childbirth,^63,65^ whereas other cultures are concerned with traditions regarding the placenta and umbilical cord which may be unable to be performed when mothers and infants are sick.^64,66^ In many cultures grandmothers care for preterm infants despite hospitalisation, and decision-making is a family affair.^65^ While, cultural and religious beliefs may support parents, they may also increase PD.^67^

### Screening for psychological distress

The overall incidence of PD in parents with an infant in NICU is high.^25,26^ Approximately 30% of mothers report clinically significant symptoms and 13% report severe symptoms^31^ which may benefit from treatment.^68^ Little is known regarding the diagnostic rate of psychiatric illness after a positive PD screen,^1^ but positive predictive values of a screen may be 12–16%, depending on the screening tool used and mental illness screened for.^69^ However, many of these mothers then require treatment.^70^ Less is known about the positive predictive value of screening tools and the predictive value in fathers, minority parents, siblings or grandparents.

Numerous screening tools are available for stress, anxiety, depression and PTSD with a wide variety of cut-off values used, even for the same screening tool.^1,6,30^ This influences the rates of PD.^31^

### Effective strategies to help parents and families with psychological distress

Intervention programmes may be supportive (providing social support and psychological counselling), educational (providing information, demonstrations, discussions, feedback) and parent-infant interaction programmes (including skin-to-skin care and infant massage).^71^

Parent support programmes, including psychological counselling, social support and psychospiritual interventions, have been shown to decrease PD and parental trauma responses.^11,31,72,73^ Cognitive behavioural therapy, including psychotherapy, has been shown to improve both maternal and paternal depression and PTSD.^12,31,72,74^ Spiritual interventions (prayers, rituals) have shown contradictory effects on parental stress, leading to decreased PD, but also in some instances leading to self-destructive behaviour.^73,75^ Parental support groups may foster peer support without increasing the burden on hospital staff,^76^ but have shown contradictory effects on stress, anxiety and depression in the short-term.^11,72,77^ Spousal support is an important source of informal support.^78^ Mindfulness and relaxation techniques may also decrease stress in parents^72^ but recorded relaxation guided imagery and other relaxation therapies have shown no effect on stress, depression or anxiety.^37,74^

Parental education and information programmes including videos, pamphlets, books, discussions (both groups and on-on-one sessions) regarding the NICU environment and activities, infant demonstrations and feedback from professionals regarding their infant. These programmes showed inconsistent effects on stress, depression and anxiety in parents.^12,14,71^ Applied formats (discussion/ demonstration sessions, written information, books, videos) varied in duration, frequency and follow-up and were often multi-pronged.^71^ In-hospital interventions, however, appear to be more effective than those provided after discharge from the NICU.^14^ Nursing interventions, including NICU orientation sessions, parent education and discussions surrounding their infant, have been shown to decrease maternal stress regarding the NICU environment and infant appearance over time but did not decrease stress regarding parental role.^11,71,78^ Fathers viewed nursing information as helpful, but if the information was not regularly updated, increased their stress.^78^

Education regarding PTSD, psychotherapy (specifically the 6-session Treatment Manual) and good neonatal care are effective for preventing and treating parental PTSD.^74^ Expressive art (making art, writing, journaling, scrapbooking)^72,74^ as well as various forms of music (non-verbal music, music therapy, singing but not music writing)^74,79–81^ may be beneficial in PTSD symptoms. These interventions may decrease PTSD symptoms, if combined with other psychological interventions (counselling^74^), but may also provide a respite for parents, as a distraction and place to connect with other parents.^72^ The use of web diaries may also be beneficial in alleviating stress symptoms.^82^

Skin-to-skin care (SSC), or kangaroo mother care (KMC), is practiced by many NICU’s internationally, to decrease neonatal mortality and morbidity, improve neonatal growth, improve breast feeding and improve infant-maternal bonding.^83^ SSC may improve maternal mood and decrease depression but may be dependent on duration and frequency of the SSC.^79,80,84,85^ For fathers, the initial SSC may be overwhelming and frightening, but may later improve infant-father interactions and overall emotional state.^15,78,86^ SSC may also improve parents’ perception of their parental role, parental competency and increase their bond with their infant.^87^ SSC has no effect on PTSD.^74^

Parent-infant interaction programmes, where parents are provided with infant education as well as taught infant-cued interactions (COPE, MITP, NIDCAP), show contradictory effects on parental stress, depression and anxiety^11,13,73^ but do lead to increased parental confidence, improved parent-infant interaction and parental knowledge of their infant’s behaviour. Maternal and paternal massage of the infant may also decrease parental anxiety.^11,15^ Various parental attachment and relationship-based interventions, including SSC, tactile stimulation, permission to change their infant’s nappy, smiling, hugging or touching their baby may decrease parental PD.^15,88^

Family centred care (FCC) or family integrated care (FIC) are methods of assisting and involving parents in the care of their infants.^71^ FCC/FIC emphasises four fundamental values: dignity and respect, information sharing, family involvement in care, and family collaboration.^89^ It seeks to incorporate families in the planning, implementation, and assessment of care and to make their perspectives as valuable as those of healthcare professionals.^90^ FCC/FIC may decrease parental stress, anxiety and depression.^90^ Infrastructure changes in the NICU may provide additional support to parents. The use of single-family rooms as compared to open bay units may increase parental involvement with their infant, increase their presence and increase parental satisfaction but has no effect on PD.^91,92^ Other infrastructure changes include sibling visiting areas, time-out spaces, private rooms for breastfeeding/ expression of breastmilk, family accommodation close to hospital, financial support, waiting rooms for family and friends, assistance with transport and food vouchers.^92^ Family involvement interventions, integrating various parent-infant involvement, parental support structures and NICU infrastructure changes, may decrease PD.^49,92,93^

Webcams have been used to facilitate parental presence for parents who may not be able to visit their infant in NICU. Webcams may improve a parent’s sense of bonding and feeling of closeness^94^ but does not affect parental PD.^95^ Various other digital technologies have also been used with variable effects on parents’ PD,^95^ including digital education material and web applications. However, continuous webcam use may also lead to increased parental anxiety.^95^

Sibling support in the NICU remains a neglected area. Although some programmes exist, their effectiveness has not been evaluated in addressing the PD of siblings. Existing programmes include sibling access to the NICU and educational books regarding their sibling in NICU and NICU itself.^55^ Continued support from a social work and volunteers to keep the sibling busy while parents visit the NICU sibling could possibly decrease distress in the whole family.^55^

Little is known about the support requirements of racial, ethnic, gender and sexual minority parents in the NICU. FCC has been shown to provide minority parents with positive experiences, thereby decreasing PD.^49^ Minority parents also require recognition and respect as parents and co-parents in the NICU to decrease stress and anxiety that may be precipitated by perceived stigma and discrimination.^49^

Most therapeutic interventions for parental PD have been performed in high resource countries,^11^ with few performed in low resource countries and it is unknown if the same interventions would be applicable in these countries.^8^ The PD pattern differences, cultural diversity and additional stressors may contribute to the need for adapted known, or alternative, therapeutic interventions in low resource settings, as few have been performed in these countries.^22^

## Discussion

The incidence of psychological distress (anxiety, depression, stress, PTSD) in all family members (mothers, fathers (irrespective of gender), siblings, grandparents), irrespective of geographical location, when an infant is admitted to NICU, is high. More than half of mothers and up to one-third of fathers suffer from one or more forms of PD. The incidence of PD in gender and sexual minority parents is poorly described, as is that in siblings and grandparents.

Various therapeutic interventions have been used to prevent and manage PD in parents with an infant in NICU. These vary widely, with most interventions showing contradictory evidence as to the effect on PD. Few interventions are available for siblings, gender and sexual minority parents and none for grandparents. The use of family-centred, combination interventions, whilst families are in the NICU, would seem more effective than single interventions or those after discharge from NICU.

The admission of an infant into NICU causes significant PD in parents. Parental PD may persist for years after the infant has been discharged^9^ and may continue to influence infant outcomes.^10^ Early recognition, diagnosis and management of family PD is therefore essential. Although PD has been well described in mothers and fathers, the incidence of PD in other minority parents, siblings and grandparents is unknown.

Various screening tests have been used for anxiety, depression, stress and PTSD in NICU. Different cut-off values for identifying parents at risk have also been used, preventing the ability for the recommendation of specific tests and diagnostic values.^8^ Screening programmes should be routinely implemented to allow for early recognition and referral, if required. Standardised, culture-specific tests,^8^ with population specific cut-off values, should be used that will enable appropriate referral. The American Academy of Obstetrics and Gynaecology perinatal mental health screening toolkit recommends the Edinburgh Postnatal Depressions Screen (EPDS) or Patient Health Questoinnaire-9 (PHQ9) for depression, General Anxiety Disorder 7 (GAD-7) screen for anxiety and Primary Care-PTSD screen (PC-PTSD) for PTSD.^96^ Despite this, few hospitals have screening programmes in place.^97,98^ Streamlining of screening tests and cut-off values may enable better interpretation of incidences of PD, across populations, and accurately interpretate the success of interventions on PD.

Most research has addressed maternal PD, whilst less is known about paternal PD. Its presentation and manifestations differ from that of mothers and this difference requires recognition and acknowledgement. The emotional support received from her partner may prevent maternal health disorders and support her recovery,^99^ but at the cost of increasing the emotional strain on the father.^100^ Maternal and paternal PD are interlinked^8^; therefore both parents should be evaluated if one parent is noticed to be emotionally distressed.

Various interventions have shown differential effects between mothers and fathers (SSC, education, information). Therapeutic interventions and management should, also, therefore be adapted to include fathers’ needs. The socio-cultural aspects and requirements of fatherhood should be considered (e.g. paternal presence only in the first few weeks in Taiwan, deprecation of sick and preterm infants in Ethiopia^101^), as these factors may need to be considered during intervention design.

Few interventions have been shown to be effective in preventing parental PD, but some have been modestly effective at decreasing PD, such as family-focused instructional interventions. Single interventions may be less effective than combinations of interventions and combination interventions should rather be implemented.^71^ Parental presence in NICU is important in relieving parental PD and should be promoted.^102,103^ Parents need to be assessed for barriers that may prevent their presence and increase PD, such as transportation, financial issues, lodging, and sibling care.^104^

Interventions may also need to be adapted for use in low resource countries. Few such studies have been performed to assess whether interventions designed for use in high resource countries would be applicable for translation and use in low resource countries.

Little is known regarding the incidence of PD in minority racial, ethnic, sexual and gender groups. Their support needs are poorly described, and few specific intervention programmes have been described. Racial and religious minorities in different countries may experience stigmatisation, as may some other groups, such as drug users.^105,106^ Increased awareness of PD in these minority groups are required and interventions may need to be adapted.

Little is known regarding the incidence of sibling PD and few interventions are available. Continued parental relationships, assurance that parental support is still available to the sibling, prevention of sibling isolation, fostering co-operation, co-ordination and sharing of experiences will ensure integration of the sibling into a difficult family situation and decrease PD effect on the sibling and family as a whole.^51^ Siblings within the NICU environment require inclusion in PD intervention programmes with their parents.

NICU medical staff are an important source of formal support and information for families.^4^ Information is a continuous requirement by parents. Information should be consistent, delivered by a single person or small group of health care workers (HCWs),^2^ delivered in a caring and empathetic style,^107^ adapted to parental understanding, preference for mode and time of communication, personal beliefs, values, cultural requirements^38^ and inclusion of other family members.^108^

Although not addressed in this review, NICU remains a stressful and often traumatic experience for parents that may lead to variable and prolonged PD.^109^ Psychological surveillance may need to continue post-NICU discharge as may interventions^110^ and include non-primary caregivers.

This review has some limitations. A meta-analysis could not be performed due to the inclusion of quantitative and qualitative data. This allowed for inclusion of detailed data but prevented statistical analysis. Although the aim was to review data regarding PD in all family members, there was only single or limited reviews available for siblings and sexual and gender minority peoples and no studies for grandparents. Due to the nature of the meta-review, various systematic reviews provided conflicting data on interventions. The diversity of screening tests, cut-off values, intervention types and duration of each, even within the same categories, as well as delivery at different time points within the NICU stay period may have contributed to these discrepancies, making the recommendation of any single intervention difficult. Family focused interventions with multiple components, including education, guided observations, support or counselling based on individual needs have been noted to be more effective in decreasing stress than specific therapies such as music therapy, or medical procedural changes.^107^ Similarly, core components of direct parental support, psychosocial support and instruction related to parent-infant interaction have been demonstrated to decrease symptoms of stress in parent-child interventions.^60^

There are numerous gaps that still require clarification within the field of family PD when an infant is admitted to NICU. Priority should be given to finding a screening tool that is appropriate for a wide spectrum of peoples and cultures and to determine appropriate cut-off values. The appropriate timing of these screening tests also needs to be determined. This would enable appropriate clinical management of family members but also improve interpretation of research data. Significant research is required into the incidence of PD in siblings, sexual and gender minority peoples and grandparents. Little is known about PD in NICU parents with pre-existing mental health disorders and they may need alternative screening tests as well as interventional strategies. The ideal intervention, or combination of interventions, is unknown. From the current review, it seems that a single intervention is less likely to decrease PD in NICU family members, and more research is required into combinations of interventions. All interventions have mostly been aimed at single participants, with few studies involving both parents. No studies have provided interventions for the entire family, including siblings and grandparents. Future research should also be aimed at interventions incorporating all family members.

## Supplementary information

Supplementary Table 1

Supplementary Table 2

Supplementary Table 3

Supplementary Table 4
