# Supplementary material for: Psychological distress in the neonatal intensive care unit: a meta-review
Source: Pediatr Res. 2024 Sep 26;96(6):1510–8. doi: 10.1038/s41390-024-03599-1 (PMC11624136; doi:10.1038/s41390-024-03599-1)
Supplement: Supplementary file 1 — Supplementary Table 1 [file 41390_2024_3599_MOESM1_ESM.docx]

| **Table 1: Summary of methodological aspects of systematic reviews included in the meta-review** | | | | | | | | | |
| --- | --- | --- | --- | --- | --- | --- | --- | --- | --- |
| **Study, year** | **Aim** | **Study type** | **Search strategy** | **Bias assessment** | **Countries included** | **Time frame** | **No studies** | **Participants** | **Heterogeneity** |
| **Aagaard , et al. 2008 ^38^** | Describe maternal experiences in NICU | Qualitative meta-ethnographic | PubMed, CINAHL , Web of Science, PsycINFO, references | NS | USA, UK, Australia, Sweden, Denmark | 2000-2007 | 14 | 159 mothers | NS |
| **Schappin, et al. 2013 ^43^** | Investigate the influence of infant, family and environmental factors on parental stress between term & preterm infants & mothers vs fathers | meta-analysis | CINAHL, EMBASE , PsycINFO , Web of Science | funnel plots | Australia, USA, Canda, UK, Norway, Malaysia, India, Japan, NZ, Turkey | up to 2011 | 51 | 2599 mothers  426 fathers | high |
| **Rosenstock et al, 2014 ^35^** | Experiences of adolescent parents in NICU | Qualitative/ quantitative | Web of Knowledge, MEDLINE, Embase, PubMed, CINAHL | NS | USA, Brazil, NZ, Turkey, Canada | 1990-2013 | 22 | 2317 parents | NS |
| **Tahirkheli, et al. 2014 ^31^** | Review of PPD in NICU | Quantitative | PubMed, Medline, and PsycINFO databases, Google Scholar | NS | NS | 1972-2013 | 23 | 2182 parents | NS |
| **Vazquez , et al. 2014 ^4^** | Awareness & practices for quality delivery of care in NICU | Qualitative | PsycINFO, ProQuest , PubMed, CINHAL | NA | USA, Canada, Australia, Taiwan, China, Sweden, Norway | 2006-2012 | 14 | 143 parents | NS |
| **Provenzi, et al. 2015 ^16^** | Review of experience of fathers in NICU | qualitative | CINHAL, ISI Web of Science, PubMed, Scopus | JBI – good quality | USA, Canada, Europe, Iran | 200-2014 | 14 | 148 fathers | NS |
| **Sisson, et al.**  **2015 ^44^** | Synthesize qualitative experiences of fathers in NICU | Qualitative | INAHL Plus, Academic Search Premier, MEDLINE ,PsycINFO | CASP-reasonable | 12 countries | 1980-2012 | 24 | 232 fathers | NS |
| **Al Maghaireh, et al. 2016 ^28^** | Review of parental experiences in NICU | Qualitative | Science Direct, PubMed, CINAHL, EBSCO, SpringerLink, Psychology & Behavioural Sciences, Collection and Web of Science | CASP-high quality | NS | 2000-2014 | 9 | 184 parents | NS |
| **Mousavi et al,**  **2016 ^75^** | Understand needs of Iranian parents in NICU | Qualitative | Iran Medex, Magiran, SID, PubMed, Scopus, and Google Scholar | COREQ-low | Iran | up to April 2015 | 16 | 211 parents | NS |
| **Beck et al,**  **2017 ^24^** | Describe PTSD in mothers & interventions in mothers in NICU | Qualitative | PubMed and CINAHL | CASP | Europe, USA, Isarel, Canada, Iran | 1981-205 | 30  5 qualitative | 2034 mothers | NS |
| **Beck et al,**  **2017 ^37^** | Integrate findings of qualitative & quantitative research of PTSD in NICU | Mixed methods | PubMed, CINAHL, PsycINFO | CASP | USA, Australia, NZ, Turkey Netherlands, India, Canada, Italy, Iran, S Korea | 2006-2015 | 37  12 qualitative 12 intervention | 2458 mothers | NS |
| **Roque, et al.**  **2017 ^1^** | Summarise evidence of mental health of parents in NICU | Qualitative/ Quantitative | CINAHL, MEDLINE, PubMed, Global Health, Cochrane Library, PsycINFO, Embase, Scopus, Web of Science, Social Work Abstracts, ProQuest, Conference Proceedings | NS | Most studies from Oceania, USA | up to 2014 | 66 articles: 9 qualitative, 2 mixed methods | 6425 parents | NS |
| **Loewenstein et al. 2018 ^21^** | Socio-ecological framework analysis of inter & intrapersonal effects of causes of PD in parents | Quantitative | PsycINFO, EBSCOhost, Academic search premier, Medline, CINAHL complete, Health Source | NS | Jordan, USA, Sweden, Italy, Taiwan, France, Germany, Korea, China, Norway, NZ, Australia, Nigeria, Turkey | 2011-2018 | 26 | 4622 parents | NS |
| **Prouhet, et al.**  **2018 ^40^** | Determine extent of psychological stress in fathers in NICU, types of stressors | Quantitative | Ovid Medline, EMBASE, Cochrane, PsycINFO, CINAHL | STROBE quality score - low | USA, NZ, Portugal, India, Japan, Canada, Israel, Argentina, Chile, Paraguay, Peru. | 1946-2016 | 15 | 863 fathers | NS |
| **Study, year** | **Aim** | **Study type** | **Search strategy** | **Bias assessment** | **Countries included** | **Time frame** | **No studies** | **Participants** | **Heterogeneity** |
| **de Paula et al. 2019^27^** | Association of preterm delivery and PPD | Quantitative | PubMed/Medline, Web of Science and PsycINFO | NS | India, Qatar, Greece, USA, Romania, Australia, Turkey, Greece, Canada, Europe, Australia, Poland, S Korea | 2008-2018 | 12 | >2 million mothers | Moderate |
| **Beck , et al.**  **2020 ^39^** | Lived experiences of fathers in NICU | Quantitative/ qualitative | PubMed, Scopus, CINAHL | CASP-moderate quality | USA, Europe, Iran, Canada, NZ, Jordan, Turkey | 2015-2020 | 21 | 649 fathers | NS |
| **Caporali, et al. 2020 ^22^** | Meta-analysis of PSS:NICU studies | Meta-analysis | PubMed, Scopus, Web of Science | Funnel plots-low | North America, Oceania, Asia, South America | 1993-2019 | 53 | 6239 parents | NS |
| **Logan, et al.**  **2020 ^50^** | Review barriers, perspectives and stresses of gay male caregivers in time spent in NICU | Narrative | PubMed, CINAHL, SCOPUS, and Google Scholar | NS | USA | NS | NS | NS | NS |
| **Citter et al.**  **2021 ^18^** | Mothers lived experiences of NICU & impact on occupation | Qualitative | CINAHL, PubMed, PsycINFO, Gender Studies Database, Sociological Abstracts, Social Services Abstract | ND | Sweden, Korea, Canada, USA, Israel, Mexico | 1997-2020 | 12 | 187 mothers | NS |
| **Staver, et al.**  **2021 ^6^** | Review experiences of maternal distress, associated variables, measurement and prevalence | Integrated | CINAHL, Medline, PubMed, PsycINFO, and SCOPUS | QATSDD- moderate quality studies | USA, Spain, Italy, Canada, Norway, Austria, NZ, | 2009-2019 | 33  3 qualitative | 2916 | NS |
| **Lee et al,**  **2023 ^41^** | Identify common experiences of Korean fathers in NICU | Quantitative/ Qualitative | PubMed, CINAHL, Web of Science, RISS ,Kmbase | NS | Korea | 1996-2023 | 15 | 1056 Fathers | NS |
| **McKeown, et al. 2023 ^26^** | Determine prevalence of PTSD in parents in NICU | Quantitative | PsycINFO, PubMed, Scopus, EMBASE, Web of Science, ProQuest Dissertations, theses, Global electronic databases | Hoy tool  Moderate | USA, Australia, UK | 1980-2021 | 7 | 526 parents | NS |
| **Nguyen et al,**  **2023 ^8^** | Determine prevalence of PPD | Quantitative | PubMed, Medline, Embase, PsycINFO, CINAHL Plus, Web of Science. | JBI-high quality, publication bias for depression | Nigeria, USA, Europe, Canada, Tirkey, Australia, South Africa, Brazil, India China, Malaysia, Korea, Mexico, Japan, | up to Nov 2021 | 79 | 98003 mothers | NS |
| **Malouf, et al.**  **2024^30^** | Review evidence of risk & protective factors for PTSD and anxiety in parents in NICU | Quantitative | Medline, Embase, PsycINFO, CINAHL, Web of Science, ResearchGate and Google Scholar; Grey literature- Ethos, ProQuest Dissertations & Theses and OpenGREY. | Selection bias high, rest low | mostly USA | up to December 2022 | 25 | 2704 mothers | high |
| **Shetty, et al.**  **2024 ^25^** | Understand parental psychological phenomena | Meta-analysis | PubMed, CINAHL, Embase, Clinical Key, Google Scholar | Newcastle Ottawa – low risk | USA, NZ, Turkey, Australia, Canada, Europe, India, Sri Lanka, Malaysia, Palestine, Brazil, Turkey, Iran | 2004-2021 | 41 | 6822 parents | high |
| **Silva et al.**  **2024 ^52^** | Evidence of impact of preterm birth on term-born siblings | Qualitative | PubMed, Scopus, EMBASE , opengrey.eu, Mednar databases, Google Scholar, reference list | CASP/JBI- moderate | Brazil, UK, Canada, USA, Sweden | Up to June 2023 | 7 | 329 siblings | NS |
| **Siva, et al.**  **2024 ^20^** | Review of stress and stressors experienced by parents in NICU in India | Quantitative/ qualitative | PubMed, EMBASE, SCOPUS, EBSCOhost, Web of Science, ProQuest, Microsoft Aca, DOAJ, Indian Citation Index, J‐Gate | JBI | India | 2011-2024 | 22 –quantitative  1 - qualitative | 1849 parents | high |
