# Supplementary material for: Psychological distress in the neonatal intensive care unit: a meta-review
Source: Pediatr Res. 2024 Sep 26;96(6):1510–8. doi: 10.1038/s41390-024-03599-1 (PMC11624136; doi:10.1038/s41390-024-03599-1)
Supplement: Supplementary file 2 — Supplementary Table 2 [file 41390_2024_3599_MOESM2_ESM.docx]

| **Table 2: Summary of results of diagnostic systematic reviews re psychological evaluation tools, incidence, risk factors and other results included in the meta-review** | | | |
| --- | --- | --- | --- |
| **Study, year** | **Psychological Tools included** | **Incidence/ Degree of PD** | **Results** |
| **Aagaard , et al.**  **2008 ^38^** | Interviews | NA | 5 metaphors - mother-baby relationships, maternal development, turbulent neonatal environment, reclaiming of maternal role, mother-nurse relationship |
| **Schappin, et al.**  **2013 ^43^** | 15 different tools used. Analysed only PSI & NICU:PSS | PSI values for prem parents may fall within normal range.  PSS:NICU fell in little to moderately stressful categories for all domains | Parents with preterm infants slightly more stress than term infants  Mothers more stress than fathers.  Moderated by BW, GA, maternal age, study design |
| **Rosenstock et al, 2014 ^35^** | PSS, PSS:NICU, STAI, BDI, interviews, SC90-R,ABA, | NA | Perceived parenting criticism, inadequate partner support, intimate partner violence, financial strain.  Variable effects of demographics on stress re infant looks/behaviour, NICU environment |
| **Tahirkheli, et al.**  **2014 ^31^** | EPDS, PDSS, CES-D, PSS:NICU, PSS, checklist 90-R, GHQ-30, BDI, PAI | PPD: mothers16-70%, fathers 36-60% | Higher risk in mothers with preterm vs term infants  Multifactorial etiology: NICU, financial, sound, light, technology stressor, psychological stressor - concerns of loss, separation, altered parental experience, limited ability to care for infant, helplessness |
| **Vazquez , et al.**  **2014 ^4^** | Interviews | NA | Various qualitative designs, all developed countries  Overarching theme of novice, advanced beginner, competent, proficient, expert - conceptualization of parenting process.  4 threads - proximity/ contact with baby, relationship with nurse, social support |
| **Provenzi, et al.**  **2015 ^16^** | Interviews | NA | 5 themes - emotional roller coaster, paternal needs, coping strategies, self-representation, caregiving engagement |
| **Sisson, et al.**  **2015 ^44^** | Interview | NA | Need for proximity, need for parental autonomy, feelings of vulnerability, need for communication, feeling excluded/isolated |
| **Al Maghaireh, et al. 2016 ^28^** | Interviews | NA | Most common themes stress-induces emotional problems, stress of hospitalisation, change in parental role/ bonding, loss of control, shattered confidence as parent, crisis, interpersonal relationships with staff |
| **Mousavi et al,**  **2016 ^75^** | Interviews | NA | Themes: Need for emotional support (cultural challenges, mental stress vs growth mental stress stimuli), need for instrumental support (economic challenges, physical irritation, need for FCC), need for spiritual support (spiritual prosperity/alienation |
| **Beck et al,**  **2017 ^24^** | SASRQ, DTS, PPS, PPQIES, PDEQ, DTS, PPQ-M, PPQ, IES-R | PTSD 14-79% | Higher in mothers with preterm infants compared to term |
| **Beck et al,**  **2017 ^37^** | interviews, PSS:NICU, DTS, PPTSD, PPQ, IES-R, SASRQ | PTSD 18-81% | Seeking connection/ building relationship, uncertainty/ disconnection from child |
| **Roque, et al.**  **2017 ^1^** | 83 different scales/ questionnaires | ASD/ PTSD 23-28%  Anxiety: mothers 24%, fathers 20%  Depression: mothers 35%, fathers 30.8% | Most studies mothers only.  Stress related most to parental role, alcohol/ drug abuse and transfer of partner due to complications. Variable effects of communication, family circumstances, socio-demographics.  Depression - differs mothers vs fathers, variable associated factors, generally not more than general population  Anxiety - possible effect of race, little consistency across studies.  PTSD & depression related, history of mental disorder, number of concurrent stressors, parental role expectations, variable effects of GA, BA, infant illness |
| **Loewenstein, et al. 2018 ^21^** | EPDS, STAI, PSS:NICU, PROMIS, CES-D, PDSS, MOIPPQ, LEC, BDI, SCID, SASRQ, IES, GHQ | Anxiety: Mothers: 12,3-51%, Fathers: 20-51%.  Stress: 52%.  Depression: mothers 19,1 -60%, fathers 30.8%-51%  PTSD; mothers 25,5-81.8%, fathers: 33%-66.7% | Variable effects of all factors  Intrapersonal characteristics - history mental illness, BW, GA, mechanical ventilation, birth trauma, preterm birth, altered parental role.  Interpersonal factors - marital status, gender differences, family cohesion.  Institutional, community & public policy - lack of information, perceived negative attitudes of medical personnel |
| **Prouhet, et al.**  **2018 ^40^** | PSS:NICU, PSS:IH, PSS, PSQ | overall moderately stressful | Prem fathers>term fathers. Higher levels in young fathers, extreme prematurity, ELBW. Changed in parental role highly stressful. Different stressors for different subscales |
| **de Paula et al.**  **2019 ^27^** | EPDS, DASS, CES-D, 2PHQ, BDI | PPD: 27-40% | PPD OR 3,16 (2,18;4,58)  PPD in 1st 6 weeks higher in mothers of in preterm infants compared to term  OR dependant on screening tool & cut-off value.  Mostly cohort studies |
| **Beck , et al.**  **2020 ^39^** | Interviews, PSS:NICU, | Stress: low-moderate degree. | 4 Themes: unfamiliar waters, responsibility to bear alone, torn between responsibilities, unexpected journey. Narrative writing, skin-to-skin decreased stress levels, parental support programme no effect |
| **Caporali, et al.**  **2020 ^22^** | PSS:NICU | Variable scores across sub-domains of NICU:PSS | Mothers higher global stress scores than fathers,  Differences between parameters (mothers more than fathers for sights/ sounds, infant behaviour).  Timing has marginal effect, effect of geographical location on sights/sounds, parental role alteration most stressful |
| **Logan, et al.**  **2020 ^50^** | NS | NA | Little data.  Legal barriers (USA), challenges in surrogacy, lack of research, lesbian nonbirth mother vs gay fatherhood, heterosexual vs gay fatherhood differences |
| **Citter et al.**  **2021 ^18^** | Interview | NA | Themes identified - struggling to claim maternal role, lack of psychological readiness to be mother, seeking connection with baby, |
| **Study, year** | **Psychological Tools included** | **Incidence/ Degree of PD** | **Results** |
| **Staver, et al.**  **2021 ^6^** | 20 different | Multiple types of distress at same time.  14-40% anxiety + depression,  82-86% depression + stress.  Depression 20-100% depending on tool  Anxiety 31-98% depending on tool,  Trauma/ PTSD 6-43% dependant on tool,  stress 20-100% dependant on tool,  12-78%Stress+depression+anxiety+PTSD | Variable effects of married/ unmarried/ co-habitation on depression and distress  Lower education, increased distance from hospital, infrequent visits increase distress and conflicting data re infant illness severity, CS and parity on distress  Increased PTSD with trauma history, antepartum hospitalisation  Preterm birth, appearance of baby, sights & sounds, altered parental role related to depression |
| **Lee et al.**  **2023 ^41^** | PSS, NPI, MAI, PAS, NFNI | Moderate stress | Highest stressor of NICU environment - change in parental role, appearance/behaviours, communication,.  Affected by older age, type of family, lack of information and type of neonatal illness, lower BW, GA. Burdened by work & house & visiting & lack of information  4 Themes: paternal stress, paternal attachment, fathers’ adaptation, fathers’ need for support |
| **McKeown, et al.**  **2023 ^26^** | PCL, IES-R, DTS | Mothers: 4.5-0-33% PTSD  Fathers: 0-33% | Mothers more PTSD than fathers,  PTSD related to NICU experience, variable association with prematurity, admission to NICU, reactions to NICU.  Prior acute stress disorder predictor of PTSD  Correlation with depression, anxiety, chronic guilt |
| **Nguyen et al.**  **2023 ^8^** | EPDS, CES-D, BDI, HADS, ICD-10, DSM-III, BDI, interviews | Maternal depression:29,2%, anxiety 37,7%.  Paternal depression 17,4%, anxiety 18,3% | Maternal depression higher in 1st month, no geographical effect.  Maternal anxiety higher in 1st month, differed between continents, length of neonatal hospitalisation.  Paternal depression higher in 1st month and differs across continents, married, multiple births.  Paternal anxiety higher in 1st month and differed across continents, multiple births.  Maternal and paternal were correlated with each other |
| **Malouf, et al.**  **2024 ^30^** | SASRQ, PLC-5, mPPQ, IES-R, ASDI, ASDS, | PTSD incidence mothers 7-55%, fathers 4-21,5% | Various scores used  62 potential risk & protective factors. Parent demographics (10), pregnancy & birth factors (7), Infant demographics (5), infant health & care factors (17), parental menta health/ trauma history (4), parental postnatal mental health history (4), parental stress, coping, support factors (11), other (4)  PTSD OR>3: Pre-existing MHD, infant illness severity, positive ASD screening, positive PHQ2 score, positive HADS score, GA, BW, maternal education, poverty, depression  Anxiety: OR>3: pre-existing mental health disorders, positive ASD screening, gestational age, CS, anxiety high, education |
| **Shetty, et al.**  **2024 ^25^** | 7 different scales | Anxiety: mothers 13-93%, fathers 0.09-46%.  Depression mothers: 18-52%, fathers 0.08-18%.  Stress (includes ASD, PTSD), mothers 23-76% ,fathers 0.06-35%. | Most participants mothers.  Worse in mothers compared to fathers.  Few studies provided cut-off scores.  Most studies performed between 1-4 weeks, not post discharge.  Most studies in high income countries |
| **Silva et al.**  **2024 ^52^** | Surveys, semi structured interviews | NA | Data collected from parents.  Reported outcomes - anxiety/ depressed, acceptance, resentment, hero/protector, reactive, withdrawn, aggressive, sleep disorders, less parental attention, developmental aggression, eating problems |
| **Siva, et al.**  **2024 ^20^** | PSS:NICU, PIP, other | Maternal stress: moderate-severe  Paternal stress; severe | Lower maternal stress in breastfeeding. Variable effects for maternal age, education, occupation, income, birth order, family type, religion, number of children, days hospitalised.  Highest stressor was relationship with baby and parental role. high heterogeneity.  Paternal stress associated with financial resources, alteration in parental role, lack of family support.  Parental stress - variable levels.  Single qualitative study - uncertainty due to communication gap, financial constraints, cultural rituals, barriers to bonding |
