# Supplementary material for: Psychological distress in the neonatal intensive care unit: a meta-review
Source: Pediatr Res. 2024 Sep 26;96(6):1510–8. doi: 10.1038/s41390-024-03599-1 (PMC11624136; doi:10.1038/s41390-024-03599-1)
Supplement: Supplementary file 3 — Supplementary Table 3 [file 41390_2024_3599_MOESM3_ESM.docx]

| **Table 3: Summary of interventional systematic reviews included in meta-review** | | | | | | | | | |
| --- | --- | --- | --- | --- | --- | --- | --- | --- | --- |
| **Study, year** | **Aim** | **Study type** | **Search strategy** | **Country** | **Sample size** | **Reported bias** | **No studies** | **Total participants** | **Heterogeneity** |
| **Benzies, et al.**  **2013 ^71^** | Estimate the effects of early interventions on parent outcomes | Quantitative | MEDLINE, EMBASE, Cochrane Database of Systematic Reviews, CINAHL, ERIC, Web of science | USA, Europe | 1990-2011 | Cochrane collaboration tool - adequate | 18 | 3431 parents | moderate |
| **Athanasopoulou, et al. 2014 ^80^** | Review of KMC on maternal mood & infant interaction | Qualitative | (ASC; EBSCO), AMED ,EBSCO), BMJ Journals Collection, Cochrane Library, EBSCO, PsycINFO, PubMed, ScienceDirect, and Web of Science | UK, Colombia, Australia, Israel, Taiwan, Italy, Brazil, India, South Korea, USA | up to 2012 | NS | 13 | 731 mothers | NS |
| **Chertok, et al.**  **2014 ^11^** | Effectiveness of interventions aimed at decreasing NICU-related maternal stress | Qualitative | National Guideline Clearinghouse, PubMed, CINAHL, EBSCOhost. | USA, Canada, UK, Germany, Norway, Italy, Australia, Turkey, Netherlands | 1998-2014 | NS | 17 | 1830 parents | NS |
| **Tahirkheli, et al.**  **2014 ^31^** | Review of PPD in NICU | Quantitative | PubMed, Medline, and PsycINFO databases, Google Scholar | NS | 1972-2013 | NS | 23 | 2182 parents | NS |
| **Beck et al.**  **2017 ^37^** | Describe PTSD in mothers & interventions in mothers in NICU | Qualitative | PubMed and CINAHL | CASP | Europe, USA, Isarel, Canada, Iran | 1981-205 | 30 | 2034 mothers | NS |
| **Epstein et al.**  **2017 ^96^** | Effects of webcam use in parents in NICU | Integrative (MMAT) | MEDLINE, CINAHL, Web of Science, Google Scholar | USA, Sweden Singapore | up to 2016 | NS | 11 | 326 parents | NS |
| **Mendelson, et al.**  **2017 ^12^** | Effects of parent-focused NICU interventions on maternal depression & anxiety | Qualitative/quantitative | PubMed, Embase, PsycINFO, Cochrane, and CINAHL | mostly USA | up to 2015 | low | 12 | 1044 mothers | low |
| **Mirghafourvand, et al. 2017 ^13^** | Effect of COPE on parental mental health | Quantitative | Cochrane, PubMed, Scopus, Google Scholar, ProQuest, Science Direct, SID, Magiran, Iranmedex | USA & Iran. | 2000-2015 | CASP-moderate | 4 | 532 parents | Moderate to high |
| **Ding, et al.**  **2019 ^91^** | Review of effects of family centred Care | Meta-analysis | Medline, CINAHL, Embase, PsycINFO, BNI,AMED, Cochrane Database of Systematic Reviews, Chinese databases China National Knowledge Infrastructure (CNKI), Wanfang Data | China, Australia, NZ, Canada, Taiwan, USA, Iran, Sweden, UK | up to 2018 | Cochrane – moderate/ high bias | 19 | 2248 parents | High |
| **Hunt, et al.**  **2019 ^76^** | Review evidence of peer support groups | Qualitative/quantitative | Medline, Embase, PsycINFO, Social Policy and Practice (SPP), HMIC (via OvidSP), CINAHL Complete (via EBSCOhost), BNI, PQDT, ASSIA (via ProQuest), Social Sciences Citation Index, Conference Proceedings Citation Index – Science and Social Sciences and Humanities (Web of Science, Clarivate Analytics, Cochrane Library (CDSR and CENTRAL) | Canada, USA, Finland: | up to 2018 | EPHPP – weak/ moderate | 14 | 1246 parents | NS |
| **Sabnis et al.**  **2019 ^94^** | Effectiveness of hospital interventions for parental distress | Meta-analysis | PubMed, PsycINFO, CINAHL, and SCOPUS for | North America, Europe, Iran, Asia, Australia, Brazil | up to 2017 | NS | 33 | 5887 parents | NS |
| **Klawetter, et al.**  **2019 ^81^** | Review of relationship between maternal engagement in NICU & preterm and maternal outcomes | Integrated | PubMed and PsycINFO | USA | up to 2018 | none | 33 | 2136 mothers | NS |
| **Study, year** | **Aim** | **Study type** | **Search strategy** | **Country** | **Sample size** | **Reported bias** | **No studies** | **Total participants** | **Heterogeneity** |
| **Scime, et al.**  **2019 ^85^** | Effect of skin-to-skin on PPD mothers | Meta-analysis | CINAHL, Cochrane Library, EMBASE, MEDLINE (Ovid), PsycINFO, and PubMed | South Korea, Iran, France, Brazil, Israel, USA, UK | 1979-2017 | Cochrane Collaboration Tool/ Robins low | 7 | 435 mothers | high |
| **Gibson, et al.**  **2020 ^95^** | Review research exploring impact of webcams on parental attachment | Qualitative | PubMed, ProQuest (PsycINFO), Medline, MedNar, CINAHL, EMCARE, Scopus, Web of Science, and Google Scholar | CCAT- moderate | no dates | CCAT | 3 | 87 parents | NS |
| **Mu, et al.**  **2020 ^88^** | Evidence regarding experiences of parents providing KMC to preterm infants in NICU | Qualitative | PubMed, CINAHL | Sweden, South Africa, Denmark, USA, Norway. | 1970-July 2018 | JBL | 9 | 107 parents | NA |
| **van Veenendaal, et al. 2020 ^92^** | Effect of open bays vs family rooms on parents | Meta-analysis | MEDLINE, EMBASE, PsycINFO, the Cochrane Central Register of Controlled Trials (CENTRAL), Web of Science, Clinicaltrials.gov, ICTRP | USA, Turkey, Sweden, Norway, Europe | up to November 2019 | serious | 17 | 909 parents | high |
| **Cong et al.**  **2021 ^86^** | Effect SSC on maternal anxiety, stress, PPD | Meta-analysis | PubMed, Embase, Cochrane Library, PsycINFO, CINAHL, Web of Science, CNKI, WanFang Database and Sinomed | 6 countries - China, Iran, UK, USA, Israel, Turkey | 2006-2020 | Cochrane collaboration tool – low/uncertain | 8 | 728 mothers | Moderate |
| **Filippa, et al.**  **2021 ^15^** | Determine type of paternal interventions prior NICU discharge, positive impacts, differences between mother & father | Qualitative | INAHL, Cochrane Central Register of Controlled Trials, Embase, PubMed and PsycINFO | Canada, Columbia, Germany, Iran, Italy, Lebanon, Norway, Korea, Swede, USA. | 1995-2020 | none | 14 | 539 parents | ns |
| **Ocampo, et al.**  **2021 ^72^** | Psychosocial interventions or support programmes to reduce psychological in NICU fathers | Qualitative | EBSCOHOST | USA, Greece, Australia, Taiwan, UK. | 2006-2019 | none | 7 | 1130 fathers | NS |
| **Zhang et al.**  **2021 ^79^** | Effectiveness of empowerment programs parental mental health problems | Quantitative | PubMed, Cochrane Library, EMBASE, Web of Science, CINAHL, PsycINFO, China Biology Medicine (CBM), China National Knowledge Infrastructure (CNKI), VIP, and Wanfang Database | USA, China, Iran, Taiwan, Colombia | Up to March 2020 | high | 8 | 1029 parents | NS |
| **Dahan, et al.**  **2022 ^77^** | Review of peer support groups in NICU | Qualitative | MEDLINE, ISI Web of Science, Cochrane Database, and Google Scholar. | USA, Canada | 2011-2021 | NA | 9 | 1245 | NA |
| **Holm, et al.**  **2022 ^78^** | Evidence to support fathers of preterm infants & effectiveness on paternal well-being | Integrative | PubMed (MEDLINE), Embase, CINAHL, PsycINFO, Cochrane, Scopus, Web of Science, SweMed+, and ProQuest Dissertation & Thesis Global. | Iran, Sweden, Taiwan, USA, Spain, Turkey, Denmark, Netherlands, France, Norway, Canada | 2010-2021 | MMAT - no data | 18 | 871 fathers | NS |
| **Kim et al.**  **2022 ^89^** | Components of attachment and relationship-based interventions for preterm infant & families in NICU | Meta-analysis | PubMed, MEDLINE, Embase (OVID), PsycINFO, CINAHL Cochrane Database | USA, UK, Taiwan, India, Switzerland, Netherlands, Sweden, Australia, South Korea, Ireland | 1999-2021 | Cochrane Collaboration Tool - low | 10 | 1268 mothers | high |
| **North et al.**  **2022 ^93^** | Effect of FCC on families | Qualitative | PubMed, Embase (Elsevier), (EBSCO), and World Health Organization Global Index Medicus | 15 MIC (China, Iran, India), HIC (Sweden, Canada, NZ, Australia, Taiwan, South Korea, USA) | 1972-Aug 2021 | Cochrane tool | 15 | 5240 parents | low to high |
| **Laccetta, et al.**  **2023 ^74^** | Review of interventions to prevent/ treat PTSD in parents NICU | Integrated | MEDLINE, Scopus, and ISI Web of Science | North America, Europe, Western Aisa | until 2022 | NIH Pre-Post tool - low | 15 | 1053 parents | NS |
| **Study, year** | **Aim** | **Study type** | **Search strategy** | **Country** | **Sample size** | **Reported bias** | **No studies** | **Total participants** | **Heterogeneity** |
| **Pathak et al.**  **2023 ^87^** | Effect of KMC on parents mental and physical health | meta-analysis | Cochrane Central Register of Controlled Trials, Cochrane Register of Studies Online, PubMed®, Web of Science, Scopus and EMBASE | Australia, Brazil, China, Colombia, Germany, Ghana, India, Islamic Republic of Iran, Israel, Italy, Malawi, Nigeria, Republic of Korea, Sweden, Türkiye, United Kingdom, United Republic of Tanzania, United States | up to January 2023 | Cochrane tool/ ROBINS-I: low to high | 30 | 7719 parents | high |
| **Brelsford, et al.**  **2024 ^73^** | Psychospiritual interventions provided to parents in the NICU and/or post discharge. | integrated | PubMed, PsycINFO, and Cochrane Library (Cochrane Database of Systemic Reviews, Cochrane Central Register of Controlled Trials | USA, Iran, Turkey | 2013-2023 | CASP - Uncertain | 4 2 studies during NICU stay | 205 mothers (NICU studies) | NS |
| **Yinger, et al.**  **2024 ^49^** | review to understand experiences of LGBTQ parents in NICU | Integrated | EBSCOhost, ProQuest, and Web of Science | NS | up to Sept 2023 | none | 6 | 9 LGBTQ mothers | NA |
