# Supplementary material for: Psychological distress in the neonatal intensive care unit: a meta-review
Source: Pediatr Res. 2024 Sep 26;96(6):1510–8. doi: 10.1038/s41390-024-03599-1 (PMC11624136; doi:10.1038/s41390-024-03599-1)
Supplement: Supplementary file 4 — Supplementary Table 4 [file 41390_2024_3599_MOESM4_ESM.docx]

| **Table 4: Summary of therapeutic systematic reviews included in meta-review** | | | | | |
| --- | --- | --- | --- | --- | --- |
| **Study, year** | **Timing of intervention/s** | **Psychological tool** | **Intervention** | **Numerical results** | **Results** |
| **Benzies, et al.**  **2013 ^71^** | Varied per intervention | STAI, NS | Promotion of parental awareness of child/ interpretation of behavior/ response to behavior, provided advice/anticipatory guidance/ emotional support, increased parental awareness of their influence on child, promoted parental well-being through decreased stress | stress SMD -0.04 (-0.23;0.15), anxiety SMD -0.54 (-0.95;-0.12), depression SMD-0.39 (-0.57;-0.20) | Various interventions - parental support, parental awareness, increase parental awareness, promote parental well-being. Psychosocial support better outcomes for mothers. Meta-analysis significant decrease in stress, anxiety, depression |
| **Athanasopoulou, et al. 2014 ^80^** | 20-60 minutes-24 hours, daily-3xweek, 2-4 weeks | PSS:NICU, STAI, EPDS, PSI-SF, VAMS, interviews, MAI, CES-D | Kmc | none | KMC variable effects on maternal mood |
| **Chertok, et al.**  **2014 ^11^** | Variable duration & frequency dependant on type of intervention | PSS:NICU, CES-D, EDPS, BDI-II | Information/ education/ empowerment, , Parent-infant interaction programmes massage, nursing interactions, psychological interventions, peer support | NS | Variable effect of interventions - differed by timing & intensity |
| **Tahirkeli et al,**  **2014 ^31^** | Not specified - overview of interventions | NS | Educational & psychosocial (cognitive behaviour therapy, COPE, journal writing, problem-solving education), family-based and provider interventions (nurse-led interventions, family intervention programme | NS | COPE decreased stress compared to controls & cost effective, journal writing decreases stress, problem-solving education less depression at follow-up, nurse education (video, education, tour) decrease anxiety re sights/ sounds |
| **Beck et al.**  **2017 ^37^** | Variable | PSS:NICU | Educational, paternal presence, narrative writing, paternal skin-to-skin, parent-centred communication support | NS | Educational programme and narrative writing decreased stress, paternal skin-to-skin increased stress, individualised communication no effect |
| **Epstein et al,**  **2017 ^96^** | when parents not in NICU, specific times, real time | PSS:NICU, STAI, qualitative interviews | Skype, facetime, angel eye, NIC view, Webcams | NA | Parents stress may increase with webcam use, qualitative interviews suggest variable effects on anxiety |
| **Mendelson, et al.**  **2017 ^12^** | Daily-weekly, single session - 15-55min sessions | BDI, BDI-II, STAI, CES-D, EPDS, QIDS, | Various interventions (CBT, educational approaches, maternal-infant responsiveness training | depression SMD -0.16 (-0.32;-0.00), anxiety -0.12 (-0.29;0.05), | CBT improved depression, combination and education-based did not. No effect on anxiety. Longer duration better than short |
| **Mirghafourvand, et al. 2017 ^13^** | NS | NS | Cope | Stress -1.72 (-1.97;-1.47)  Anxiety -1.01 (-1.48;-0.53) | Statistical decrease in maternal stress after 2nd phase CPE, , significant decrease in state anxiety after 1st phase. No evaluation depression as different phases (significant heterogeneity) |
| **Ding, et al.**  **2019 ^91^** | Varying components and varying durations | SDS, SAS, STAI, PSS:NICU, PSI-SF, BDI-II, VASS | COPE, PBIP, FCC, CBIP, NDMT,FIC, PPN | none | Decreased stress and anxiety but mixture of time points & tests, heterogenic interventions, most developed countries |
| **Hunt, et al.**  **2019 ^76^** | variable time after birth, variable times per week6-12 weeks, 1:1 or groups | Interviews, POMS, STAI, PSS | Support groups | none | Reductions in perceived stress, anxiety  Better support, sharing & understanding. Lower psychological outcomes at discharge and months (variable) after DC |
| **Sabnis et al,**  **2019 ^94^** | Variable depending on intervention | PSS:NICU, PSS, STAI, PPSDQ, Davidson trauma scale | Support groups, parental caregiving interventions, single rooms, FIC, diaries, acupuncture, SSC narrative writing massage, education, MITP, PBIP, NIDCAP, psychological support, Cues & Cares COPE, books, videos, crisis intervention | changing medical care SMD -0.08 (-0.44;0.28),  complimentary medicine -0.47 (-0.83;-0.12),  family centred instruction SMD -0.37 (-0.59;-0.15),  psychotherapy -0.37 (-0.59;-0.15) | Complementary medicine and family centred instruction decreased distress but not changing NICU medical care |
| **Klawetter, et al.**  **2019 ^81^** | Variable depending on type of intervention | Interviews, | Maternal engagement-related activities: NICU visitation, skin-to-skin or kangaroo care holding, traditional holding, infant massage, music stimulation, and interventions designed to facilitate maternal-infant interaction/bonding, psychoeducation, combinations | none | Variable effects of KMC on maternal mental health |
| **Scime, et al.**  **2019 ^85^** | daily, 3xweek, 10 sessions 1-2 hours | EPDS, GHQ, CES-D, BDI, PPDS, EPDS, | KMC | PPD SMD -1.04 (-1.30;-0.79) | Varied designs  Substantial heterogenicity of interventions.  5 different depression tools  Overall, no effect of ssc |
| **Gibson, et al.**  **2020 ^95^** | Variable usage | STAI, PSS:NICU | Web cameras | NS | Exploratory studies, reduce worry, contradictory evidence re effect on stress & anxiety, negative=hypervigilance of parents |
| **Study, year** | **Timing of intervention/s** | **Psychological tool** | **Intervention** | **Numerical results** | **Results** |
| **Mu, et al.**  **2020 ^88^** | NS | Interviews | KMC | NA | 5 themes synthesized: sense of emptiness of parental role, barriers in translation of parental roles, preparation enhances parental role expectations, KMC enhances parental competency, encouragement/ support from family/friends |
| **van Veenendaal, et al. 2020 ^92^** | Variable starting points of FCC | PSS:NICU, DASS-21, EPDS, BDI, STAI, SPSQ | Family rooms | Stress SMD -0.41 (-0.58;-0.24)  Anxiety SMD -0.55 (-1.00;-0.10)  Depression SMD -0.15 (-0.48;0.18) | Varied times - during NICU, at discharge, after discharge. Upon discharge significant decrease in parental stress scores, generally better parental outcomes in family rooms |
| **Cong et al,**  **2021 ^86^** | 2-120 min, daily-3xweek | STAT, PSI, PSS:NICU, GHQ | KMC | Anxiety: SMD -0.72 (-1.08;-0.35)  Stress: -0.84 (-1.59; -0.09) | Decreased anxiety but dependant on length per day and frequency,  Significantly decreased stress. |
| **Filippa, et al.**  **2021 ^15^** | 5min touch, 30s stimulation 3060 min SSC daily 3xweek, free interaction, | STAI, PSS:NICU, PSI-SF | KMC, tactile interaction, speech | . | Decreased anxiety and stress |
| **Ocampo, et al.**  **2021 ^72^** | Variable frequency dependant on intervention | NS | Mindfulness, Psychotherapy based, expressive art-based, peer-based support groups | NS | Qualitative & quantitative studies reported. Variable effects on stress, anxiety and depression |
| **Zhang et al,**  **2021 ^79^** | Variable, or NS | STAI, PSS:NICU, BDI-II, PSI-SF, PSS | COPE, educational CD, empowerment education, psychological support, combinations | overall SMD -0.56 (-86;-0.25), stress SMD -0.82 (-0.97;-0.67), anxiety SMD -3.52 (-5.42;-1.62), depression SMD -0.30 (-0.54;-0.05) | Decreased anxiety and stress but not depression. Education showed significant benefit above COPE. In-hospital studies more effect than after discharge |
| **Dahan, et al.**  **2022 ^77^** | Variable depending on intervention | Interview | Peer support, parent education/ parents involved in assessment by resource parent | NA | 653 activities in 47 types of initiatives. Decreased stress & overall anxiety, depression |
| **Holm, et al.**  **2022 ^78^** | Variable duration and frequency of sessions  First 5 days, 30-60 min, 3 sessions  Day of admission – 15 days – until discharge | PSS:NICU, PSS”IH, ISRA, BDI, EPDS, EPDS, STAI, PBQ, MPAS | Education, SSC, empowerment, psychological support, video nursing dialogue, single family rooms | none | Helped interaction between baby & father, Information decreased stress, anxiety, nursing support interventions mixed effects on fathers |
| **Kim et al,**  **2022 ^89^** | Variable duration & frequency  45-240 min per day, 1-3 sessions | STAI, POMS, PSS:NICU, MPAS | Free play, SSC, talking, feeding, smiling, hugging, changing nappy | maternal anxiety MD -1.26 (-5.24; 2.66), PSS:NICU SMD -0.15 (-0.36;0.08), maternal depression MD -0.64 (-0.83;-0.44), maternal traumatic stress MD -0.33 (-0.61;-0.06). | Attachment/ relationship interventions decrease maternal distress (traumatic stress and depression) |
| **North et al,**  **2022 ^93^** | Variable interventions, starting points and duration | STAI, PSS:NICU | FCC incl bedside care, medical decision-making involvement, infrastructure changes, developmental/ behavioural care, SSC, infant massage, psychosocial support, transport vouchers, home transitioning, FIC, PPIP, | NICU:PSS MD -0.29 (-0.56; -0.01, I291%),  maternal anxiety STAI MD -1.79 (-3.11;-0.48, I1 0%) | Low/moderate quality evidence - Family involvement interventions may decrease maternal anxiety & stress |
| **Laccetta, et al.**  **2023 ^74^** | Variable duration and frequency | PC-PTSD, DTS, PPQ, IES-R, P-SS,DTS | Video recordings, cognitive restructuring, relaxation, mindfulness, parent-infant interactions, auditory/tactile/visual stimulation, SSC, expressive writing/ trauma narrative, nonverbal music. Infant definition, NICU education, PTSD education | Pre/post intervention scores SMD -0.92 to -2.1 | PTSD prevention - combination of therapies best, |
| **Pathak et al,**  **2023 ^87^** | Variable duration, variable days per week | Interviews, PSS:NICU, BDI, PSI-SF, EPDS, GHQ, MABS, VAMS, MAI, STAI, CES-D, PPQ, PSS | KmMC | Maternal stress (SMD: −0.82; 95% CI: −1.32 to −0.32)  anxiety (SMD: −0.62; 95% CI: −1.01 to −0.23),  depression RR 0.76 (0.59;0.96), SMD -0.22 (-0.47;0.02),  distress MD -4.71 (-9.77;0.35) | Reduced stress, anxiety |
| **Study, year** | **Timing of intervention/s** | **Psychological tool** | **Intervention** | **Numerical results** | **Results** |
| **Brelsford, et al.**  **2024 ^73^** | 30-60 min | PSS:NICU, STAI, MI:PS, CES-D, WHO-QOL-Bref | Various interventions (prayer, religious objects, photos, DVD, discussions, sessions re religious self-care, varying length and frequency, variable outcome measures | NS | Lower stress levels post-intervention |
| **Yinger, et al.**  **2024 ^49^** | NA | Interview | FCC | NA | Aspects to co-parent not being treated as family and not allowed to see infant (variable practices at hospitals), respectful treatment, heterosexist assumptions/discrimination, seeking inclusivity/respect. Collaboration/empowerment |
